# The progressive fragmentation of the KIT/PDGFRA wild-type (WT) gastrointestinal stromal tumors (GIST)

**DOI:** 10.1186/s12967-017-1212-x

**Published:** 2017-05-23

**Authors:** Margherita Nannini, Milena Urbini, Annalisa Astolfi, Guido Biasco, Mara A. Pantaleo

**Affiliations:** 1 Department of Specialized, Experimental and Diagnostic Medicine, Sant’Orsola-Malpighi Hospital, University of Bologna, Via Massarenti 9, 40138 Bologna, Italy; 2 “Giorgio Prodi” Cancer Research Center, University of Bologna, Bologna, Italy

**Keywords:** GIST, SDH, Quadruple wild-type, ETV6–NTRK3, MEN1, MAX, FGFR1

## Abstract

Recent advances in molecular biology have revolutionized the concept of KIT/PDGFRA wild type (WT) gastrointestinal stromal tumors (GIST) than the past. Indeed, from being defined as GIST without KIT or PDGFRA mutations, we are now faced with the opposite scenario, where KIT/PDGFRA WT GIST are “positively” defined according to their specific molecular alterations. In particular, if until recently KIT/PDGFRA GIST without abnormalities of KIT, PDGFRA, SDH, and the RAS signaling pathway were referred as *quadruple* WT GIST, today also this small subset of GIST is emerging out as a group of heterogeneous distinct entities with multiple different molecular alterations. Therefore, given this still growing and rapidly evolving scenario, the progressive molecular fragmentation may inevitably lead over the time to the disappearance of KIT/PDGFRA WT GIST, destined to be singularly defined by their molecular fingerprint.

Gastrointestinal stromal tumors (GIST) that lack KIT or platelet-derived growth factor receptor alpha (PDGFRA) mutations, that are around 10–15% of all cases, have always been classified as KIT/PDGFRA wild type GIST, short-named WT GIST [1]. Only recently, a more comprehensive molecular analysis have shown that KIT/PDGFRA WT GIST are a rather heterogeneous group of different diseases than one single entity [2].

20–40% of all KIT/PDGFRA WT GIST are succinate dehydrogenase complex (*SDH)*-*deficient* GIST, recognized by the loss of subunit B (SDHB) protein expression most often due to germ-line and/or somatic loss-of-function mutations in any of the four SDH subunits (A, B, C, or D). The SDH-*deficient* GIST share a pathognomonic profile characterized by young age, female gender predilection, gastric localization, mixed epithelioid and spindle cell morphology, diffuse KIT and ANO1 (DOG1) IHC positivity, frequent lymph node metastatic involvement, and an indolent behaviour even often metastatic up-front [3–9]. Moreover, *SDH*-*deficient* GIST are characterized by the over-expression of the insulin growth factor 1 receptor (IGF1R) [10, 11]. Finally, they also display a common epigenomic background, distinguished by a distinctive hypermethylation and miRNA profile [12–17]. In particular, SDH-*deficient* GIST present a marked hypermethylation profile, generally implicates the Krebs cycle as SDH-mutant paraganglioma and pheochromocytoma (Pgl/Pheo) [12]. Moreover, SDH-*deficient* GIST also show a distinctive miRNA expression profile characterized by miR-139-5p, 455-5p and let-7b signature, that may represent the epigenetic modulator of IGF1R expression and then a potential onco-miR mark of this subset of GIST [17].

The subgroup of the remaining KIT/PDGFRA WT GIST, but not SDH-*deficient*, have been further characterized: 4–13% carry a BRAF V600E mutation, are localized more frequently in small intestine and seem to have a more favourable prognosis [18–21]. Within the not SDH-*deficient*, some GIST have a neurofibromatosis (NF) type 1 mutation and show a female prevalence, a frequent non-gastric site and multifocal localization often unveiling an unrecognized NF1 syndromic condition [22–26].

Half of the KIT or PDGFRA WT GIST have been identified to be either SDH-deficient or BRAF/RAS/NF1 mutated, but the other half has still remains orphan of a specific molecular event and thus has been named as *quadruple* WT-GIST [27]. However, the transcriptome profile of this small subgroup is so profoundly different from the other GIST, either KIT/PDGFRA WT or -mutated GIST, that *quadruple WT* GIST could represent another unique group within the family of GIST [28].

Nevertheless, recently it has been shown that *quadruple* WT GIST have a greater molecular heterogeneity, with many different and probably mutually exclusive mutational events (Fig. 1). The presence of an ETV6-NTRK3 gene fusion has been firstly described in a case of rectal *quadruple* WT GIST [29]. The same translocation have been also reported in a colon primary *quadruple* WT GIST [30]. Moreover, two fusion genes involving FGFR1 were reported in three cases of *quadruple* WT GIST (FGFR1–HOOK3 and FGFR1–TACC1) [30]. Recently, other fusion events (KIT–PDGFRA, MARK2-PPFIA1 and SPRED2-NELFCD) have also been detected [31, 32]. Finally, relevant somatic mutations, including TP53, MEN1, MAX, CHD4, FGFR1, CTDNN2, CBL, ARID1A, BCOR and APC were also identified [30–33]. Interestingly, MEN1 and MAX mutations, along with NF1 and SDH, further extend the list of genes detected in KIT/PDGFRA WT GIST, genes which are characteristic for neuroendocrine tumors. Moreover, detection of high expression level of genes involved in the neural commitment process, such ASCL1 and EPHA4, further support the hypothesis of a neuroendocrine like signature for some *quadruple* WT GIST [32].

**Fig. 1:**
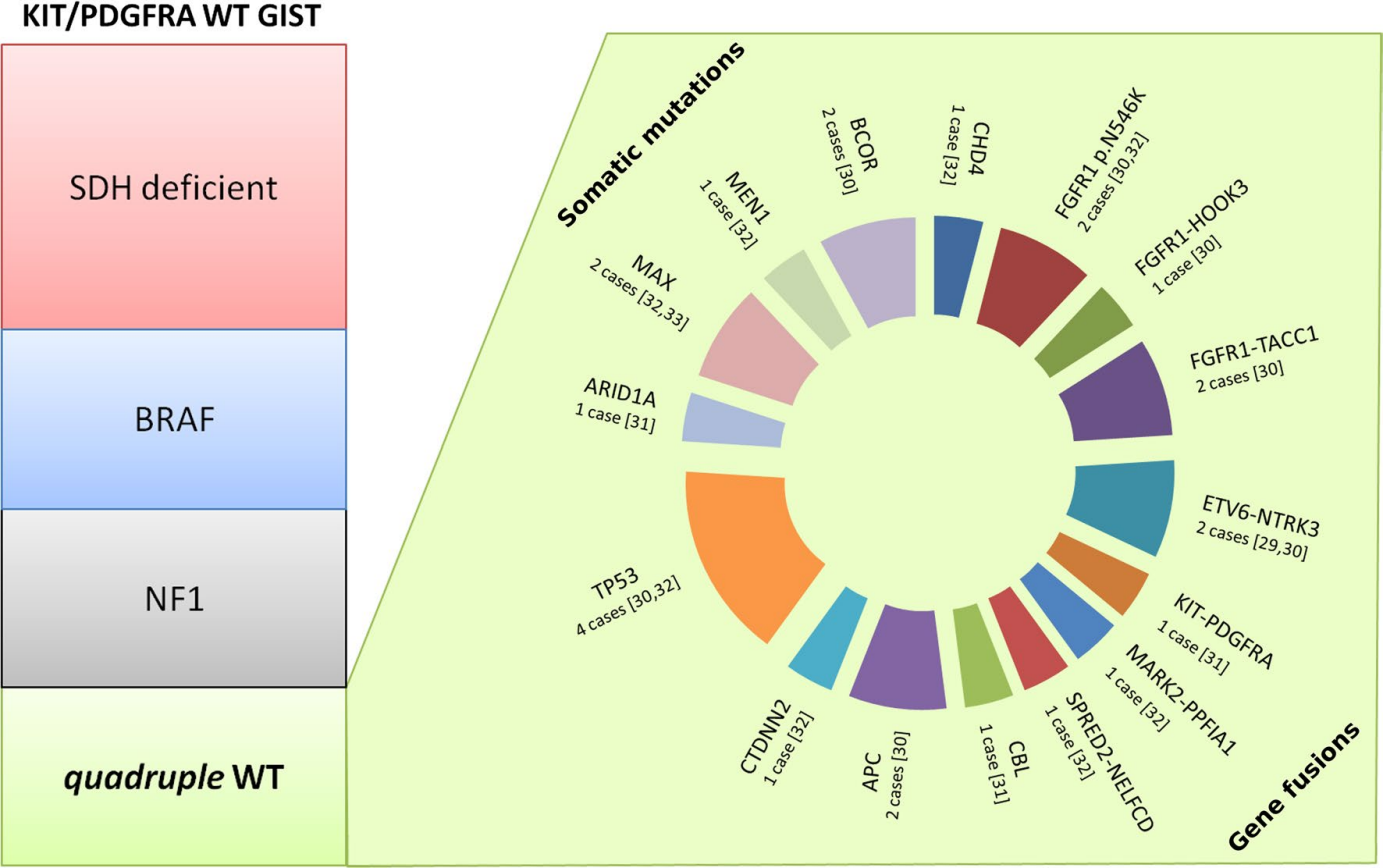
The WT GIST’s kaleidoscope

Given the recent findings, also *quadruple* WT GIST, the small subset of GIST that lack abnormalities of KIT, PDGFRA, SDH, and the RAS signalling pathway, can be considered as a group of heterogeneous single entities with different molecular alterations. Therefore, a different scenario than expected is emerging. Despite their unquestionable GIST morphology, given this marked molecular heterogeneity, *quadruple* WT GIST could be a different disease than GIST. Otherwise, trusting unquestionable GIST morphology, it could be argued that *quadruple* WT GIST may arise from a distinct population of pluripotent interstitial cells of Cajal (ICC) [34, 35], or that they may share a molecular driver at the epigenomic level, given their homogeneous gene expression profile.

If in the past KIT/PDGFRA WT GIST has been for long “negatively” defined by exclusion, we are now faced with the opposite scenario, where KIT/PDGFRA WT GIST are “positively” defined according to their specific molecular alterations. Over the time, this is inevitably leading to a progressive fragmentation of the KIT/PDGFRA WT GIST, until make them disappearing.

## Abbreviations

GIST: gastrointestinal stromal tumors
PDGFRA: platelet-derived growth factor receptor alpha
WT: wild type
NF1: neurofibromatosis type 1
ICC: interstitial cells of Cajal
SDH: succinate dehydrogenase
