# Erratum to: The progressive fragmentation of the KIT/PDGFRA wild-type (WT) gastrointestinal stromal tumors (GIST)

**DOI:** 10.1186/s12967-017-1228-2

**Published:** 2017-06-02

**Authors:** Margherita Nannini, Milena Urbini, Annalisa Astolf, Guido Biasco, Maria A. Pantaleo

**Affiliations:** 1 Department of Specialized, Experimental and Diagnostic Medicine, Sant’Orsola-Malpighi Hospital, University of Bologna, Via Massarenti 9, 40138 Bologna, Italy; 2 “Giorgio Prodi” Cancer Research Center, University of Bologna, Bologna, Italy

## Erratum to: J Transl Med (2017) 15:113 DOI 10.1186/s12967-017-1212-x

In the original version of this article [1], published on 23 May 2017, the name of author ‘Maria A. Pantaleo’ was wrongly displayed.

Originally the author name has been published as:

- Mara A. Pantaleo

The correct author name is:

- Maria A. Pantaleo

The original publication of this article has been corrected.
